# Comparative Transcriptional Profiling of Primed and Non-primed Rice Seedlings under Submergence Stress

**DOI:** 10.3389/fpls.2016.01125

**Published:** 2016-07-28

**Authors:** Saddam Hussain, Hanqi Yin, Shaobing Peng, Faheem A. Khan, Fahad Khan, Muhammad Sameeullah, Hafiz A. Hussain, Jianliang Huang, Kehui Cui, Lixiao Nie

**Affiliations:** ^1^National Key Laboratory of Crop Genetic Improvement, MOA Key Laboratory of Crop Ecophysiology and Farming System in the Middle Reaches of the Yangtze River, College of Plant Science and Technology, Huazhong Agricultural UniversityWuhan, China; ^2^College of Resources and Environment, Huazhong Agricultural UniversityWuhan, China; ^3^Shanghai Biotechnology CorporationShanghai, China; ^4^Key Laboratory of Agricultural Animal Genetics, Breeding and Reproduction, Huazhong Agricultural UniversityWuhan, China; ^5^Faculty of Agriculture and Natural Sciences, Abant Izzet Baysal UniversityBolu, Turkey; ^6^Department of Agronomy, University of AgricultureFaisalabad, Pakistan

**Keywords:** direct-seeded rice, germination, seed priming, submergence stress, transcriptome analysis

## Abstract

Submergence stress is a limiting factor for direct-seeded rice systems in rainfed lowlands and flood-prone areas of South and Southeast Asia. The present study demonstrated that submergence stress severely hampered the germination and seedling growth of rice, however, seed priming alleviated the detrimental effects of submergence stress. To elucidate the molecular basis of seed priming-induced submergence tolerance, transcriptome analyses were performed using 4-day-old primed (selenium-Se and salicylic acid-SA priming) and non-primed rice seedlings under submergence stress. Genomewide transcriptomic profiling identified 2371 and 2405 transcripts with Se- and SA-priming, respectively, that were differentially expressed in rice compared with non-priming treatment under submergence. Pathway and gene ontology term enrichment analyses revealed that genes involved in regulation of secondary metabolism, development, cell, transport, protein, and metal handling were over-represented after Se- or SA-priming. These coordinated factors might have enhanced the submergence tolerance and maintained the better germination and vigorous seedling growth of primed rice seedlings. It was also found that many genes involved in cellular and metabolic processes such as carbohydrate metabolism, cellular, and metabolic biosynthesis, nitrogen compound metabolic process, transcription, and response to oxidative stress were induced and overlapped in seed priming treatments, a finding which reveals the common mechanism of seed priming-induced submergence tolerance. Taken together, these results may provide new avenues for understanding and advancing priming-induced responses to submergence tolerance in crop plants.

## Introduction

Direct seeding of rice is increasingly being adopted in both irrigated and rainfed areas because it reduces required energy and labor and improves resource use efficiency and system productivity (Liu et al., 2015). Rice is primarily planted by direct seeding in Australia, Europe, USA, Brazil, Chile, Cuba, and other Caribbean countries (Kaur et al., 2015). This method is also becoming popular in many Asian countries. Dry seeding is a common practice in rainfed lowlands, uplands, and flood-prone areas, whereas wet seeding is extensively practiced in irrigated areas (Rao et al., 2007). However, poor germination and stand establishment of direct-seeded rice, particularly under unfavorable environmental conditions, remains a major impediment to large-scale adoption (Liu et al., 2014). In submergence-prone areas, rice farmers commonly encounter flooding after seeding, which decreases germination and seedling vigor due to the high sensitivity of rice to anaerobic conditions during germination (Miro and Ismail, 2013). Kamolsukyunyong et al. (2001) reported that approximately 15–20 million hectares of lowland rainfed rice cultivation area in South and Southeast Asia is prone to flooding. Moreover, in irrigated areas, unleveled soil surfaces or heavy rainfall after seeding may also result in submergence conditions. The uneven distribution of rainfall and extreme weather events in recent years due to climate change has increased the frequency and intensity of floods (Coumou and Rahmstorf, 2012).

A high germination rate and vigorous seedling growth are desirable traits for rice under submergence stress (Miro and Ismail, 2013). However, submergence tolerance during germination is relatively rare in rice. In an evaluation of the submergence tolerance of over 8000 gene bank accessions and breeding lines, Angaji et al. (2010) identified only five lines (0.06%) with a reasonably high level of tolerance. Although rice seeds can germinate in submerged conditions, they fail to develop roots and leaves (Miro and Ismail, 2013). Some progress has been made in unraveling the phenotypic and metabolic processes that are associated with submergence tolerance. These include fast germination, coleoptile elongation, high carbohydrate catabolism in germinating seeds, anaerobic respiration to sustain energy supply, and maintenance of the cellular extensibility of the growing embryo (Ismail et al., 2009; Miro and Ismail, 2013). Ismail et al. (2009) reported that under submergence stress, faster germination, and superior growth of tolerant genotypes was linked with a greater ability to use stored starch reserves due to higher amylase activity and higher rates of ethylene production at the germination and seedling stages. Under submerged conditions, the ethylene produced in rice plants triggers the *Sub1* gene and down-regulates shoot elongation to preserve energy for survival (Miro and Ismail, 2013). The identification of the *Sub1* locus and the elucidation of its role in the adaptation of rice to submergence was an important advance. In addition with submergence stress, the protective role of *Sub1* against other abiotic stresses, such as drought and oxidative stress, has also been reported (Jung et al., 2010; Mustroph et al., 2010). Recently, comparative analyses by Fukao et al. (2011) revealed that *Sub1A* expression was enhanced by drought and oxidative stress upon desubmergence. They further reported that *Sub1A* played a key role in water relations, detoxification of reactive oxygen species (ROS), and stress-inducible gene expression during drought. It serves as a convergence point between submergence and drought response pathways, allowing the rice plants to survive under both extremes of precipitation (Fukao et al., 2011). Nevertheless, the ability of rice seeds to germinate under anoxia cannot be explained in terms of *Sub1* genes (Alpi and Beevers, 1983). For example, the rice cultivars “M202” and “Nipponbare,” which both lack the *Sub1A* gene, exhibited greater germination under anoxia than “FR13A,” which contains the *Sub1A* gene (Xu et al., 2006; Magneschi and Perata, 2009), although other ethylene response factors (*ERFs*) may play a role in submergence tolerance at the germination stage.

Modern rice varieties are sensitive and poorly adapted to submerged conditions during germination and early growth stages (Miro and Ismail, 2013). The development of improved tolerant varieties is challenging. Although steady progress has been made, bottlenecks continue to be identified. Therefore, alternative strategies for enhancing submergence tolerance in rice, particularly at the germination and early seedling growth stages, are required for the success of direct-seeded rice in rainfed and flood-affected areas. Seed priming has emerged as an effective, short-term and pragmatic approach for increasing seed germination and the growth of rice seedlings, particularly under unfavorable environmental conditions (Khaliq et al., 2015; Hussain et al., 2016a,b; Zheng et al., 2016). Seed priming is a controlled hydration technique that induces pre-germination metabolism without allowing radicle emergence (Chen and Arora, 2013; Hussain et al., 2015). Higher germination and vigorous seedling growth after seed priming primarily occurs due to a reduction in the lag time of imbibition (Brocklehurst and Dearman, 2008), accumulation of germination-enhancing metabolites (Khaliq et al., 2015; Zheng et al., 2016), metabolic repair during imbibition (Hussain et al., 2015), osmotic adjustment (Bradford, 1986; Khaliq et al., 2015), changes in gene expression through chromosomal alterations (Chen and Arora, 2013), and increased synthesis and mobilization of proteins, starches, and lipids (Job et al., 1997). Chen and Arora (2013) proposed that seedling emerged from primed seeds cope with environmental stresses by vigorous head-start or/and cross tolerance. During priming, early imbibition process promotes the efficient mitochondrial development by augmenting energy metabolism, while after rehydration of primed seeds, main cellular processes such as the *de-novo* synthesis of nucleic acids and proteins, ATP production, activation of DNA repair (chromatin remodeling and small RNAs), and antioxidant mechanisms are triggered leading to higher stress tolerance ability (Chen et al., 2001; Li et al., 2005; Varierf et al., 2010).

Seed priming to enhance the tolerance against various abiotic stresses including drought, salinity, chilling, and heavy metals in various plant species has been studied extensively (Farooq et al., 2009; Jisha et al., 2013; Paparella et al., 2015). Hasanuzzaman and Fujita (2011) documented that selenium (Se) priming enhanced the drought tolerance in rapeseed by regulating the activities/levels of enzymatic and non-enzymatic antioxidants. Moreover, Se-priming protected the rapeseed seedlings from cadmium-induced oxidative stress by enhancing the antioxidant defense and methylglyoxal detoxification systems (Hasanuzzaman et al., 2012). Seed priming with salicylic acid (SA) was found to alleviate the negative effects of salinity stress on *Vigna radiata* through increased activities of antioxidant enzymes (Khan et al., 2014). Singh and Usha (2003) reported that SA-treated wheat seedlings exhibited higher moisture content, dry matter accumulation, antioxidant activity, and total chlorophyll content under drought stress compared to the untreated control. Recently, in a series of studies on chilling stress, we found that higher germination and seedling growth of rice after Se- and SA-priming was due to higher starch metabolism and metabolite synthesis, enhanced respiration rate, better membrane integrity and increased activities of antioxidants (Hussain et al., 2016a,b; Wang et al., 2016).

However, studies regarding the role of seed priming in submergence tolerance of rice have been limited. Ella et al. (2011) have found that seed priming improved the flooding tolerance of rice by enhancing seedling survival, carbohydrate metabolism, and antioxidant enzyme activities. A comprehensive understanding of the responsible genes and molecular mechanisms through which primed rice seedlings respond to submergence stress is of great importance to plant biology. In the present study, we evaluated the two seed-priming techniques (Se-priming, and SA-priming) under submergence stress, and carried out the transcriptome analyses to obtain greater insight into the genes and mechanisms responsible for seed priming-induced submergence tolerance in rice. To the best of our knowledge, this is the first transcriptomic study regarding the role of seed priming under submergence stress in rice.

## Materials and methods

### Plant material

Seeds of a widely grown *Indica* inbred rice (Oryza sativa L.) cultivar, Huanghuazhan were used in the present study. The initial germination and initial seed moisture content (MC) of the seeds were >95 and 9.40% (on dry weight basis), respectively. To minimize contamination during priming, seeds were surface sterilized with 2.63% NaOCl solution (household bleach diluted 1:1 with sterile water) for 30 min and rinsed three times with sterile distilled water.

### Experimental details

In order to examine the role of seed priming in alleviating the adverse effects of submergence stress in rice, different seed priming approaches viz., hydropriming, osmopriming, redox priming, chemical priming, and hormonal priming were tested in preliminary studies. Based on germination and seedling growth data, chemical priming with selenium (60 μM selenium) and hormonal priming with salicylic acid (100 mg L^−1^ salicylic acid) were the most effective treatments for enhancing submergence tolerance in rice (data not shown), and were used in further experiments. The treatments in the present study were (1) no priming + no submergence control (NP + Cn), (2) no priming + submergence stress (NP+Sub), (3) selenium priming + submergence stress (Se+Sub), and (4) salicylic acid priming + submergence stress (SA+Sub). Seeds were primed in the dark at 25°C for 24 h with constant gentle agitation. The ratio of seed weight to solution volume (w/v) was 1:5. The priming solution was renewed after 12 h (Hussain et al., 2015) and pH was adjusted at near-neutral. After 24 h of priming, the treated seeds were washed with distilled water for 2 min, surface-dried using blotting paper, and transferred to an air-drying oven at 25°C for 48 h to reduce the moisture content.

Twenty healthy seeds from each treatment were sown on two layers of filter paper in transparent plastic cups (15 cm deep, 9 cm upper inside diameter, and 7 cm lower inside diameter). To impose submergence stress, plastic cups were filled with 13 cm of distilled water; wet filter paper was used for the control treatment. The cups were placed on steel racks in a growth chamber with a 12-h light period and a temperature of 30°C day:25°C night. Humidity was maintained at 60% throughout the study. All treatments followed a completely randomized design and were replicated six times.

### Evaluation of rice seed germination, seedling growth, and starch metabolism

Seed germination was recorded on a daily basis according to (AOSA, 1990) till constant count. The shoot and root lengths of 10 randomly selected seedlings from each replication were measured at 7 days after sowing (DAS). The roots and shoots of the seedlings of each replicate were dissected, and their fresh weights were recorded immediately. The seedling vigor index was calculated by multiplying the final germination percentage by the seedling length.

Starch metabolism in rice seedlings was accessed at 5 DAS in terms of α-amylase activity and total soluble sugar content. To determine α-amylase activity, 1.0 g of fresh seedling sample was ground, mixed with 100 mL of distilled water, and incubated for 24 h at 4°C. The enzyme activity was determined from the supernatant liquid by the dinitrosalicylic acid (DNS) method (Bernfeld, 1955). To determine total soluble sugars, a ground seedling sample (1 g) was mixed with 10 ml of distilled water and incubated for 24 h at 25°C (Lee and Kim, 2000). The mixture was filtered with Whatman No. 42 (Whatman plc, Kent, UK), and the total volume was adjusted to 10 mL with distilled water. The total soluble sugars were determined by the phenol sulfuric method (Dubois et al., 1956).

Data for the rice germination and seedling growth are presented as the mean ± standard error (*SE*) of six replicates. Analyses were performed using the software Statistix 9.0 (Analytical Software, Tallahassee, FL, USA), and the mean variance of the data was analyzed using the least significant difference (*LSD*) test at the 0.05 probability level.

### RNA extraction

To obtain deeper insight into the molecular mechanisms involved in seed priming-induced submergence tolerance, transcriptome analyses were performed. Total RNA was isolated from the 4-day-old rice seedlings using an RNAprep pure Plant Kit (TIANGEN Biotech, Beijing, China). Each treatment was represented by two biological replicate shoot samples, and each sample contained shoots from at least 40 seedlings. RNA quality was characterized by agarose gel electrophoresis and by analysis with a NanoDrop ND1000 spectrophotometer (NanoDrop Technologies, Wilmington, DE, USA) and was further assessed by the RIN (RNA Integrity Number) value of 9.5 using an Agilent 2100 Bioanalyzer (Santa Clara, CA, USA).

### Library construction, and sequencing

Eight cDNA libraries were constructed using an mRNA-Seq Sample Preparation Kit (Cat# RS-930-1001, Illumina Inc., San Diego, CA; Illumina) following the manufacturer's instructions. Briefly, poly-(A) mRNA was isolated from the total RNA of each sample using Magnetic Oligo (dT) Beads. The mRNA was then fragmented into small pieces using an RNA fragmentation kit (Ambion). Using these short fragments as templates, the first cDNA strand was synthesized using random hexamer primers and reverse transcriptase (Invitrogen), and the second-strand cDNA was synthesized using DNA polymerase I and RNase H.

The cDNA fragments were purified using a QIAquick PCR extraction kit (Qiagen) and resolved with EB buffer for end repair and poly (A) addition. The short fragments were then connected with sequencing adapters, and the products were subsequently purified and amplified by PCR. Libraries were prepared from a 400–500 bp size-selected fraction following adapter ligation and agarose gel separation. Quality control analysis of the sample library was performed to quantify the DNA concentration and to validate the library. After validation with an Eppendorf Mastercycler ep realplex Real-Time PCR System, the cDNA libraries were sequenced on an Illumina HiSeq™ 2500 platform. The sequencing-derived raw image data were transformed by base calling into sequence data using Illumina Pipeline Software v1.6. The prepared libraries were sequenced using Illumina HiSeq™ 2500 with up to 40 M reads per sample which generated 2 × 100 bp paired-end reads.

### Analysis of mRNA expression profiling (RNA-seq)

The clean sequencing reads were mapped to the rice RNA reference sequence (MSU release7, downloaded from http://rice.plantbiology.msu.edu/index.shtml) using FANSe 2 algorithm (Zhang et al., 2012) with the parameters −L110 −E7 −U0 −S10. The transcripts with at least five mapped reads out of eight samples were considered as reliably detected transcripts. These transcripts were further quantified using count values, which were raw counts of sequencing reads. The count values were imported into DESeq package (Anders and Huber, 2010) of R software (http://www.rproject.org) to calculate the up-/down-regulation of genes among NP+Cn, NP+Sub, Se+Sub, and SA+Sub groups. This software provides methods to product of a condition-dependent per-gene value (CDPV), and differential expression value using the negative binomial distribution and a shrinkage estimator for the distribution's variance. The up- or down-regulated genes were identified by filtering the RNA-seq data with the following cut-off:two ratio in expression level and false discovery rate (*FDR*) of less than 0.05.

A motif search was performed using BCRANK package of R software (Ameur et al., 2009). This method takes a ranked list of genomic regions as input and outputs short DNA sequences that are overrepresented in some part of the list. The algorithm was developed for detecting transcription factor binding sites in a large number of enriched regions from high-throughput ChIP-seq experiments, however, it can also be applied to any ranked list of regulatory elements. The 2000 bp sequences for associated gene promoters were given as input in BCRANK package to identify the common motifs. Some of these identified motifs were matched to known motifs in the plant transcription factor binding sites database, JASPAR CORE plants (Lenhard and Wasserman, 2002; Sandelin et al., 2004).

### Functional analysis

After getting the differential expressed transcripts (DETs), functional annotation was done based on the function and signaling pathway of the genes. In this step, the known databases mainly including AgriGO (http://bioinfo.cau.edu.cn/agriGO/) for Gene Ontology (GO) enrichment analysis and MapMan for pathway enrichment analysis (http://mapman.gabipd.org/web/guest) were used. Considering the large amount and complex branch structure of GO biological processes and pathway, a significance threshold *P*-value (< 0.05) for biological process terms was used.

### Quantitative RT-PCR

We designed qRT-PCR primers in the regions of within 1 kb upstream of 3′-end of the transcription stop site, following the rule of amplicon length within 80–120 bp, Tm:60 ± 2 and GC% = 30–70%. The qRT-PCR was conducted in a 25-μL reaction containing 2 × SYBR master mix (Sigma-Aldrich Co., LLC), 0.2 pM each primer, and 1 μL of cDNA template. The analysis of eight selected genes was performed in a 7900 HT Fast Real-Time PCR System (Applied Biosystems, Inc.) with four independent biological replications of the RNA, including the same samples of RNA used in the transcriptome analysis. The specific primers of the analyzed genes for qRT-PCR are listed in Table S1. The LOC_Os08g03290.1 (GAPDH: glyceraldehyde-3-phosphate dehydrogenase) was kept as house-keeping gene.

## Results

### Seed priming enhanced the rice germination and seedling growth under submergence stress

**Figure 1:**
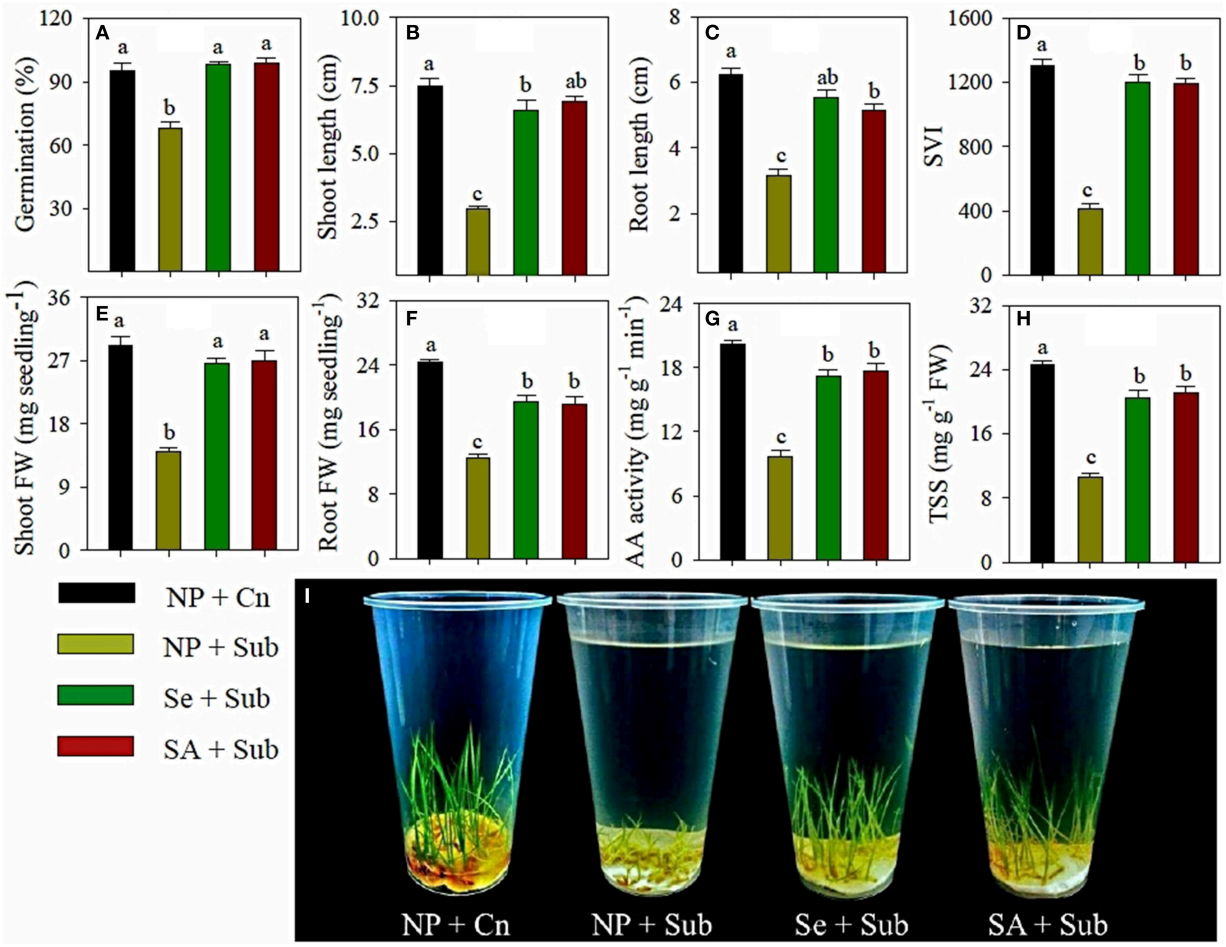
**Influence of seed priming on (A) final germination, (B) shoot length, (C) root length, (D) seedling vigor index, (E) shoot fresh weight, (F) root fresh weight, (G) α-amylase activity, and (H) total soluble sugar content of rice under submergence stress**. **(I)** Pictorial view of the primed and non-primed rice seedlings under submergence stress at 7 DAS. Data **(A–H)** represent mean ± *SE* of six replicates. Different small letters above the mean bars represents the significant difference among treatments at *LSD* < 0.05. NP, non-primed; Se, 60 μM selenium priming; SA, 100 mg L^−1^ salicylic acid priming; Sub, submergence, FW, fresh weight.

Data for the germination and seedling growth rice in response to seed priming treatments and submergence stress are presented in Figure 1. Submergence stress had deleterious effects on the germination of non-primed rice seeds; therefore, 68% seeds were germinated in NP+Sub. Seed priming was effective in alleviating the adverse effects of submergence, and germination of the rice was increased by 44 and 46% in Se+Sub and SA+Sub, respectively, compared with NP+Sub. Germination of rice in both seed priming treatments was statistically similar (*P* ≤ 0.05) to the NP+Cn (Figure 1A).

Submergence stress significantly (*P* ≤ 0.05) reduced the seedling growth of rice; maximum growth of rice seedlings was observed in NP+Cn (Figure 1). Upon exposure to submergence stress, the shoot length, root length, shoot fresh weight, root fresh weight and seedling vigor index of rice in NP+Sub were decreased by 60, 50, 52, 49, and 68%, respectively (Figure 1). However, both priming treatments ameliorated the submergence-induced growth suppression of rice. All the growth attributes were significantly enhanced in Se+Sub and SA+Sub treatments compared with NP+Sub. The seedling vigor index recorded in the Se+Sub and SA+Sub treatments was almost three times higher than that recorded in NP+Sub (Figure 1).

### Seed priming regulated starch metabolism in rice seedlings under submergence stress

The α-amylase activity and total soluble sugar content in rice seedlings varied significantly (*P* ≤ 0.05) in response to submergence stress and seed priming treatments (Figures 1G,H). The maximum α-amylase activity and total soluble sugar content were observed in NP+Cn and were reduced by 52 and 57%, respectively, in NP+Sub (Figures 1G,H). The seed-priming treatments were effective in regulating α-amylase activity and increasing the total soluble sugar contents in rice seedlings. The α-amylase activity and total soluble sugar contents in seed priming treatments were almost two times than NP+Sub, and both the seed priming treatments (Se+Sub and SA+Sub) were statistically similar to each other (Figures 1G,H).

### Transcriptome analysis of primed and non-primed rice seedlings under submergence stress

Our results (Figure 1) revealed that seed-priming treatments effectively assuaged the negative effects of submergence stress on the germination and seedling growth of rice. To underpin the molecular mechanism of Se- or SA-priming on global transcriptional profiling, all the treatments were further subject to transcriptome analysis.

**Figure 2:**
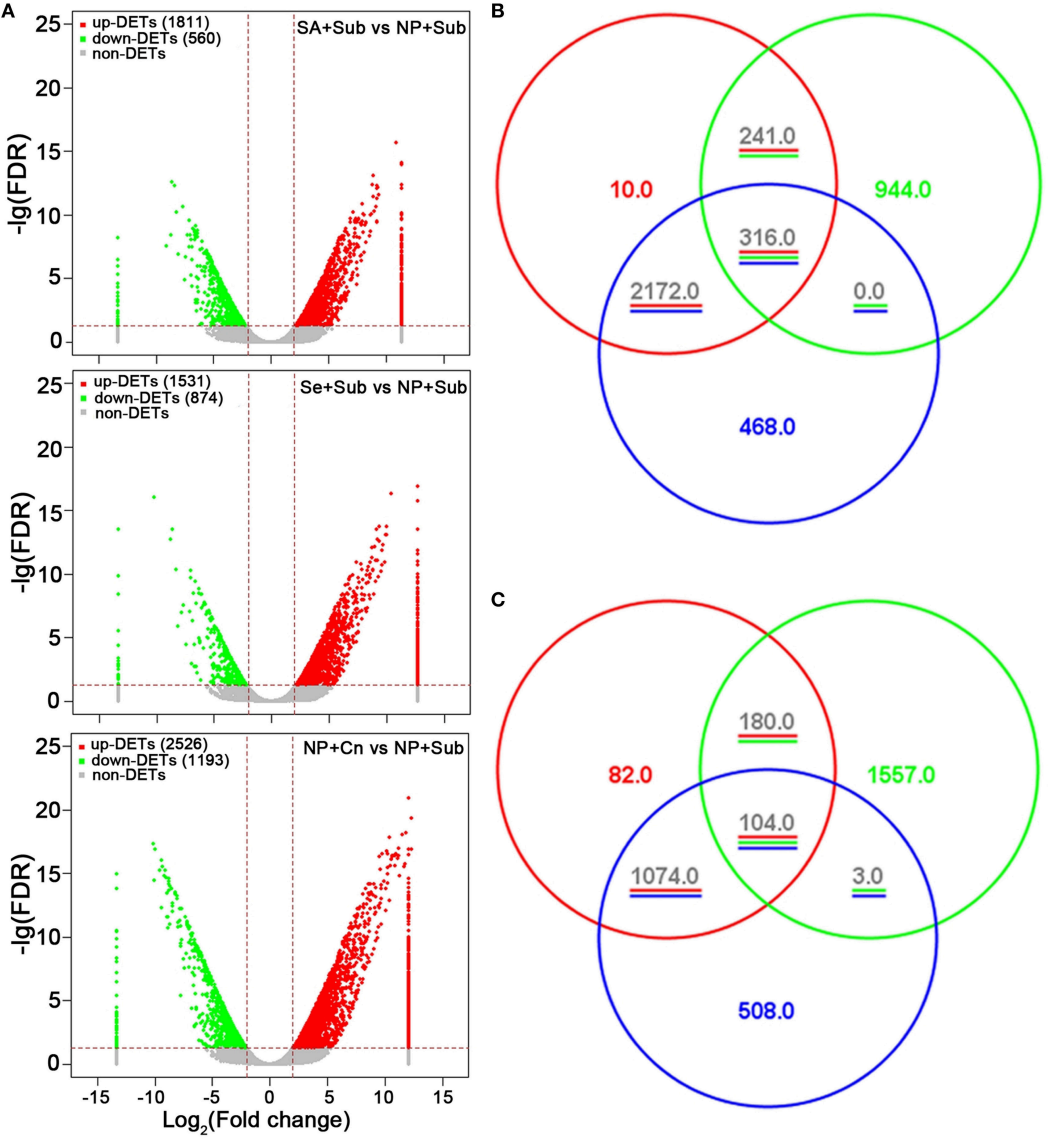
**(A)** Volcano plots of SA+Sub treatment vs. NP+Sub treatment, Se+Sub treatment vs. NP+Sub treatment, and NP+Cn vs. NP+Sub treatment transcriptomes. **(B)** Venn diagram of the up-DETs aligned into MapMan tool among SA+Sub treatment vs. NP+Sub treatment (red cycle), Se+Sub treatment vs. NP+Sub treatment (green cycle), and NP+Cn vs. NP+Sub (blue cycle). **(C)** Venn diagram of the down-DETs aligned into MapMan tool among SA+Sub treatment vs. NP+Sub treatment (red cycle), Se+Sub treatment vs. NP+Sub treatment (green cycle), and NP+Cn vs. NP+Sub (blue cycle).

The libraries were sequenced using the Illumina Hi-seq 2500 Genome Analyzer platform with paired-end 100 base-pair tags to a depth of 36,765,558–53,740,514 million reads. These reads were then mapped to the Rice transcript database. Approximately 31–46 million reads were mapped to this transcript sequence, accounting for 84.1–85.4% of the total reads (Table 1). More than 28 thousand transcripts were detected by RNA-seq, which represented more than 43% of total transcripts assembled in rice MSU databases (Table 1). Using fold change >2 and false discovery rate (*FDR*) < 0.05 as thresholds, 2371 transcripts (1811 up-regulated and 560 down-regulated) by Se+Sub, 2405 transcripts (1531 up-regulated and 874 down-regulated) by SA+Sub, 3719 transcripts (2526 up-regulated and 1193 down-regulated) by NP+Cn, were identified as differentially expressed genes (DETs) compared with NP+Sub (Figure 2A; Table S2). A list of DETs commonly expressed in both seed priming treatments is given in Table S3, while top 10 enriched common regulatory elements from 1628 DETs promoters (due to seed priming treatments) are presented in Table S4. Both Se and SA priming treatments not only depicted almost a similar DET number, but also have common regulatory elements, 90.7% DET promoters have TATA box, 74.9% have Basic helix-loop-helix factors (bHLH), 39.5% have CAAT box, and 37.5% have GC box. These results suggested that both seed priming treatments caused the transcriptional changes and were effective in alleviating the adverse effects of submergence stress. Nevertheless, higher number of DETs in NP+Cn compared with Se+Sub or SA+Sub (Figure 2) corresponds with the higher seedling growth under normal conditions (Figure 1).

**Table 1:** **Summary of RNA-seq data**.

**Sample name**	**Raw reads**	**Mappable reads**	**Mapping ratio (%)**	**Transcript number^a^**
NP+Cn (R_1_)	42,062,288	35,774,552	85.1	30,821
NP+Cn (R_2_)	36,765,558	31,124,543	84.7	30,089
NP+Sub (R_1_)	44,113,122	37,409,430	84.8	28,520
NP+Sub (R_2_)	44,099,180	37,233,227	84.4	28,506
Se+Sub (R_1_)	47,984,450	40,354,579	84.1	31,640
Se+Sub (R_2_)	44,467,438	37,438,648	84.2	31,253
SA+Sub (R_1_)	53,740,514	45,434,813	84.5	31,256
SA+Sub (R_2_)	38,552,664	32,936,215	85.4	30,191

a *The transcripts with at least five mapped reads were considered as reliably detected transcripts*.

*R_1_ and R_2_ indicate two biological replicates*.

### Pathway annotation and enrichment analysis

The DET data were submitted to the MapMan tool to align with the public protein database, and 3709, 2363, and 2400 transcripts were located in at least one point in plant biological pathways for NP+Cn, Se+Sub, and SA+Sub treatments, respectively. Pathway enrichment analysis revealed that the DETs between the NP+Cn and NP+Sub were involved in secondary metabolism, signaling, protein, development, transport, lipid metabolism, and miscellaneous functions (Table 2). The Se-priming was found to be involved in secondary metabolism, development, and cell, while SA-priming was involved in development, transport, protein, and metal handling. Development pathway showed enrichment in three treatments including NP+Cn, Se+Sub, and SA+Sub (Table S5), which would be the evidence for the better germination and seedling growth as described in Figure 1. Cell pathway and metal handling pathway were the specific enrichments in Se and SA priming treatment, respectively, suggesting that these treatments might have opted different mechanisms in enhancing rice germination and seedling growth under submerged conditions.

**Table 2:** **Overview of pathway enrichment analysis by MapMan**.

**Pathway description**	**Elements**	***P*-value^a^**
**NP+Cn vs. NP+Sub**		
Misc	500	1.39E-08
Secondary metabolism	120	1.04E-07
Signaling	209	1.97E-06
Protein	255	5.33E-06
Development	131	8.41E-05
Transport	207	0.002221
Lipid metabolism	93	0.030061
**Se+Sub vs. NP+Sub**		
Secondary metabolism	69	0.001682
Development	93	0.008649
Cell	54	0.018532
**SA+Sub vs. NP+Sub**		
Development	95	3.89E-05
Transport	117	0.004438
Protein	170	0.006502
Metal handling	16	0.025307

a *Wilcoxon RanK Sum Test*.

Venn diagram (Figures 2B,C) depicted the overlapped expressed genes and specifically expressed genes in NP+Cn, Se+Sub, and SA+Sub treatments. A total of 316 up-DETs and 104 down-DETs were common in NP+Cn, Se+Sub, and SA+Sub treatments. The overlapped up-DETs and down-DETs in seed priming treatments were 241 and 180, respectively. Interestingly, SA+Sub treatment showed the most similar up-DETs or down-DETs with NP+Cn, while, Se+Sub revealed diverse up- or down-DETs (Figures 2B,C), which suggests that the other functions might be simultaneously involved in the Se-primed germinating seedlings.

**Figure 3:**
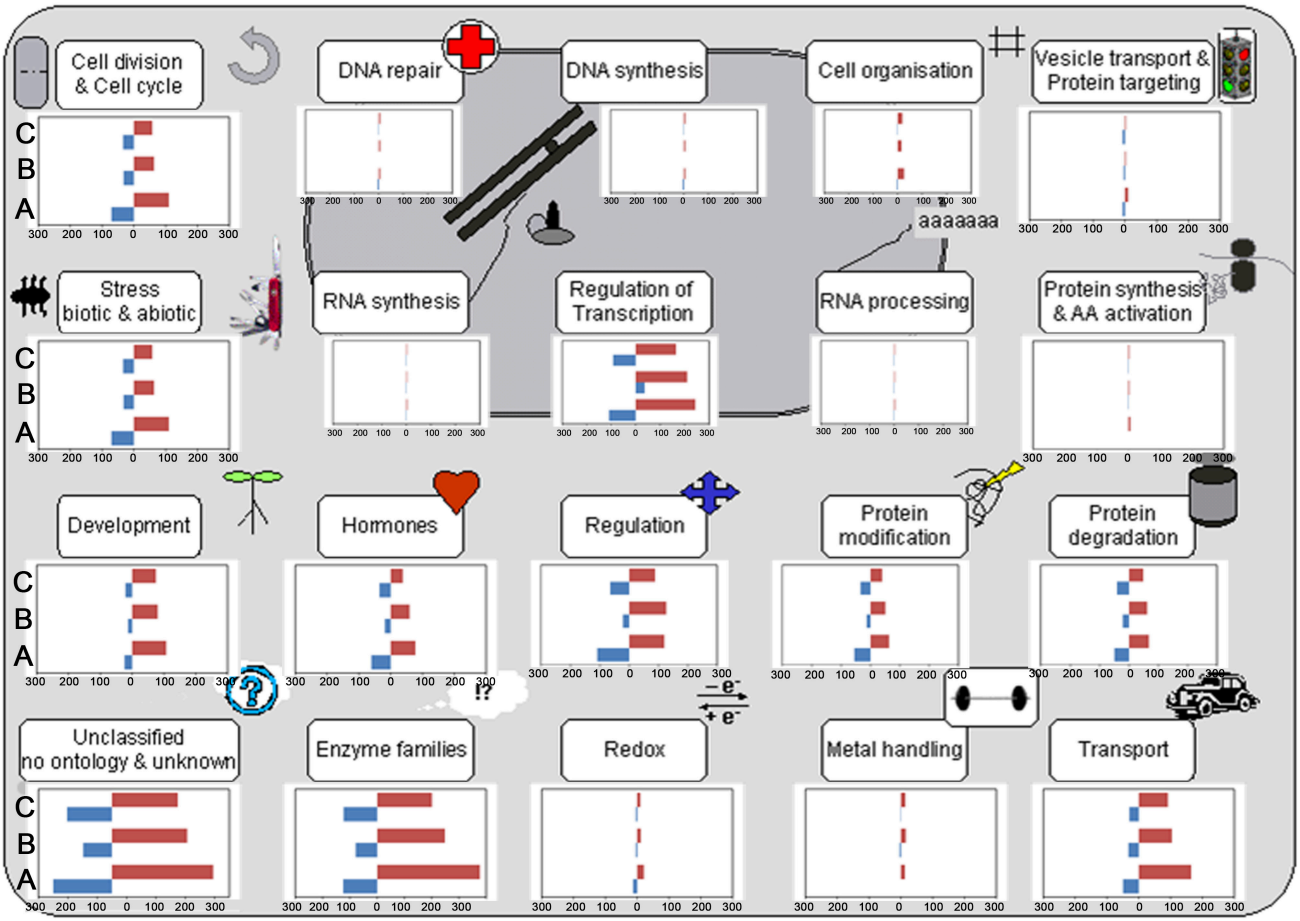
**Cell function pathway in NP+Cn (A), Se+Sub (B), and SA+Sub (C) compared with NP+Sub for rice**. Transcripts participating in the same pathway or homologs are represented by a set of closely connected cubes, and the 2-based logarithm of fold change value (log_2_FC) is denoted. Red color in bar represents the quantity of up-regulated genes, while blue denote the down-regulated genes in NP+Cn, Se+Sub, and SA+Sub.

Cell function overview pathway revealed that the most up-DETs in NP+Cn, Se+Sub, and SA+Sub treatments over NP+Sub were involved in cell cycle, cell organization, protein synthesis, development, enzymes, redox, metal handling, and transport (Figure 3). The up-DETs in NP+Cn and Se+Sub were also involved in cell division. The most down-DETs for NP+Cn treatment were involved in RNA synthesis, while both seed priming treatments depicted the most down-DETs in amino acid activation and vesicle transport.

### Gene ontology (GO) annotation and enrichment analysis

**Figure 4:**
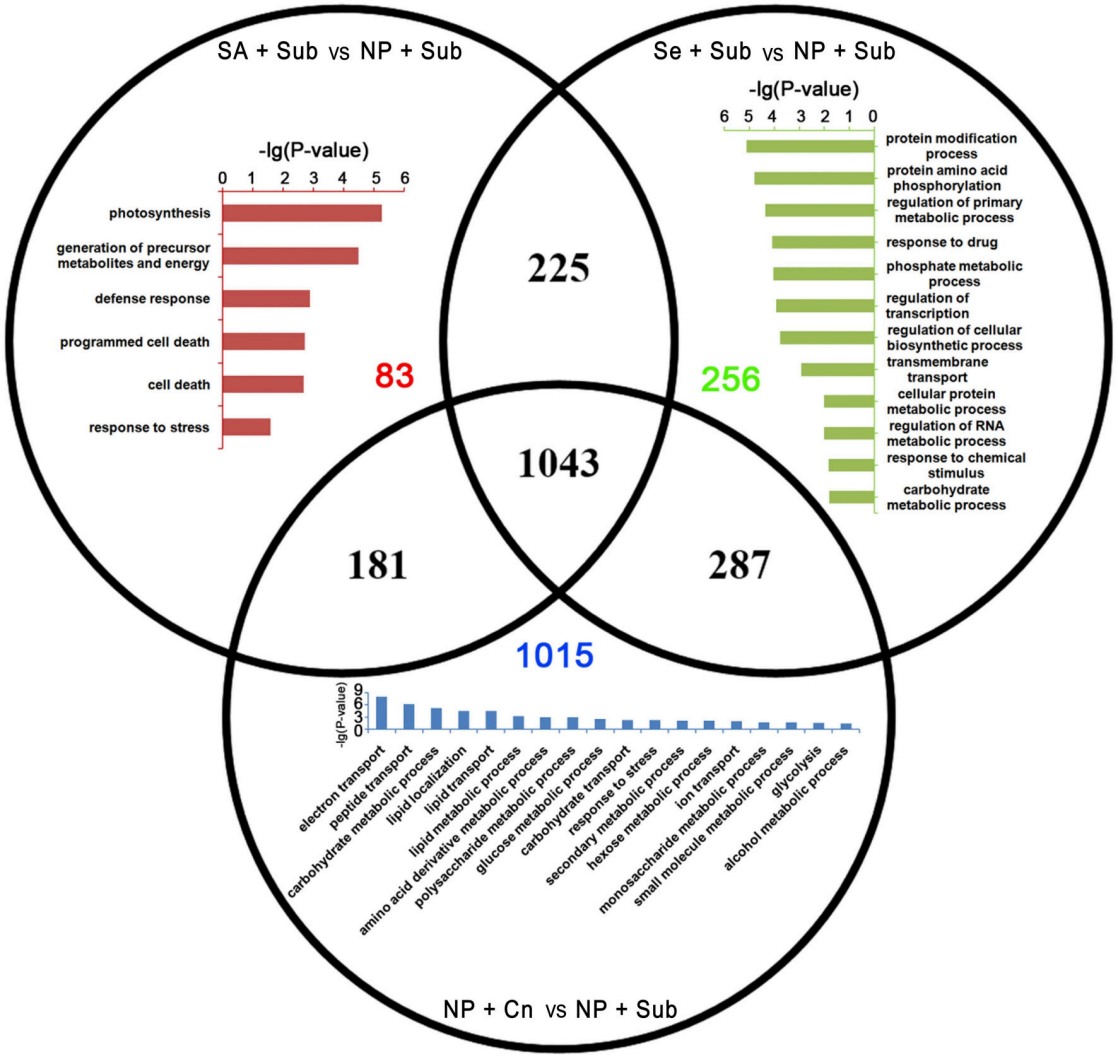
**Venn diagram of the up-DETs enriched with GO database among SA+Sub treatment vs. NP+Sub treatment (red bar diagram), Se+Sub treatment vs. NP+Sub treatment (green bar diagram), and NP+Cn vs. NP+Sub (blue bar diagram)**.

GO enrichment analysis in the biological process domain suggested that the specific up-DETs of SA+Sub were enriched with the GO terms involved in photosynthesis, energy metabolism, defense response, and programmed cell death. On the other hand, specific up-DETs from Se+Sub were enriched in protein modification process, response to chemical stimulus, regulation of transcription, and a set of metabolic processes (Figure 4).

**Figure 5:**
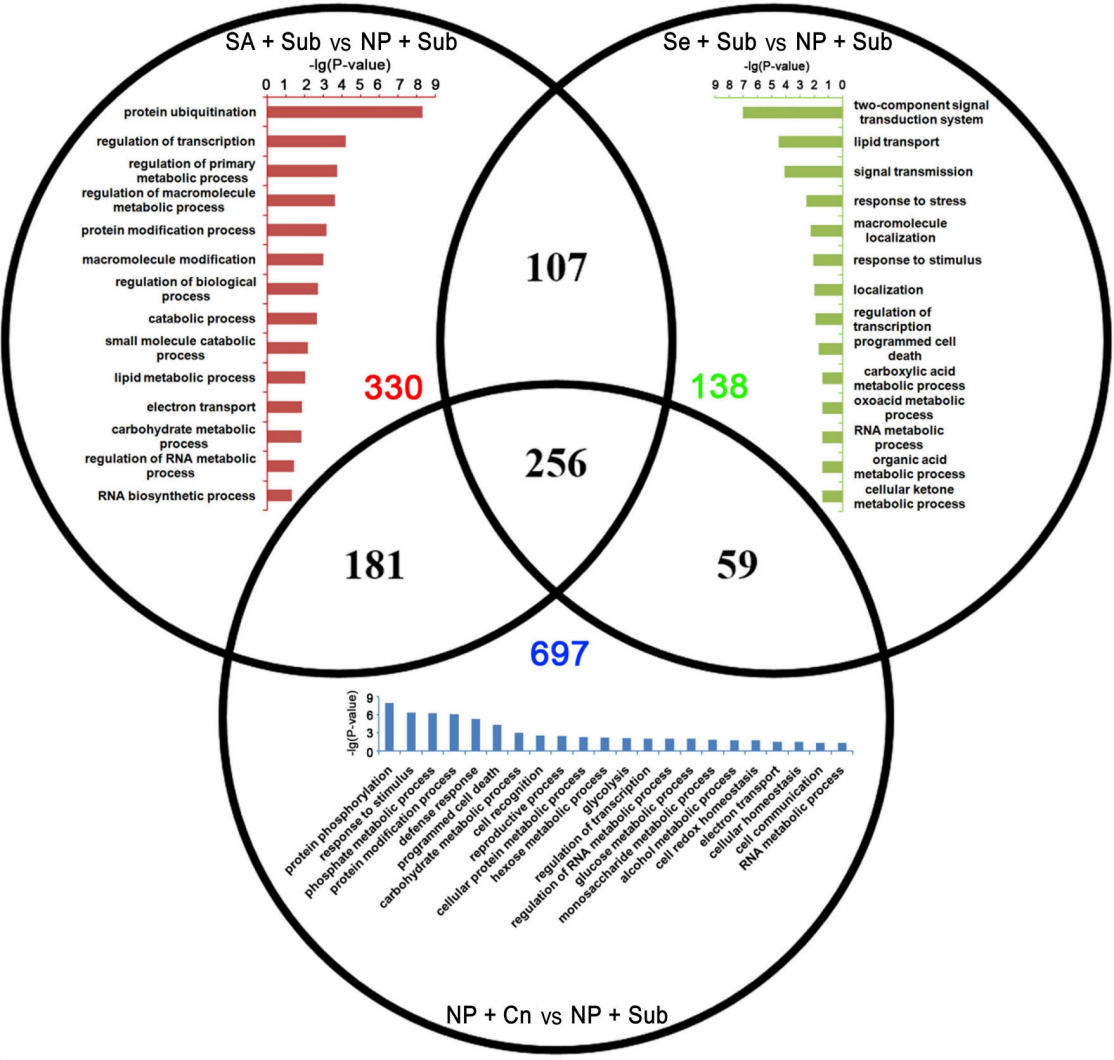
**Venn diagram of the down-DETs enriched with GO database among SA+Sub treatment vs. NP+Sub treatment (red bar diagram), Se+Sub treatment vs. NP+Sub treatment (green bar diagram), and NP+Cn vs. NP+Sub (blue bar diagram)**.

Specific down-DETs by SA+Sub were involved in gene expression regulatory and metabolism process, such as protein ubiquitination, regulation of transcription, regulation of primary metabolism. While, specific down-DETs from Se+Sub were enriched in signal transmission, response to stress, regulation of transcription, and programmed cell death (Figure 5). The SA+Sub, Se+Sub, and NP+Cn treatments showed a set of different GO terms with up-DETs or down-DETs, which might be due to the influence of submergence stress and seed priming treatments. Overlapped GO enrichments in seed priming treatments showed that many genes involved cellular and metabolic processes such as carbohydrate metabolism, nitrogen compound metabolic process, cellular, and metabolic biosynthesis, transcription and response to oxidative stress were highly up-regulated by seed priming treatments compared with NP+Sub (Table S6).

### qPCR validation

**Figure 6:**
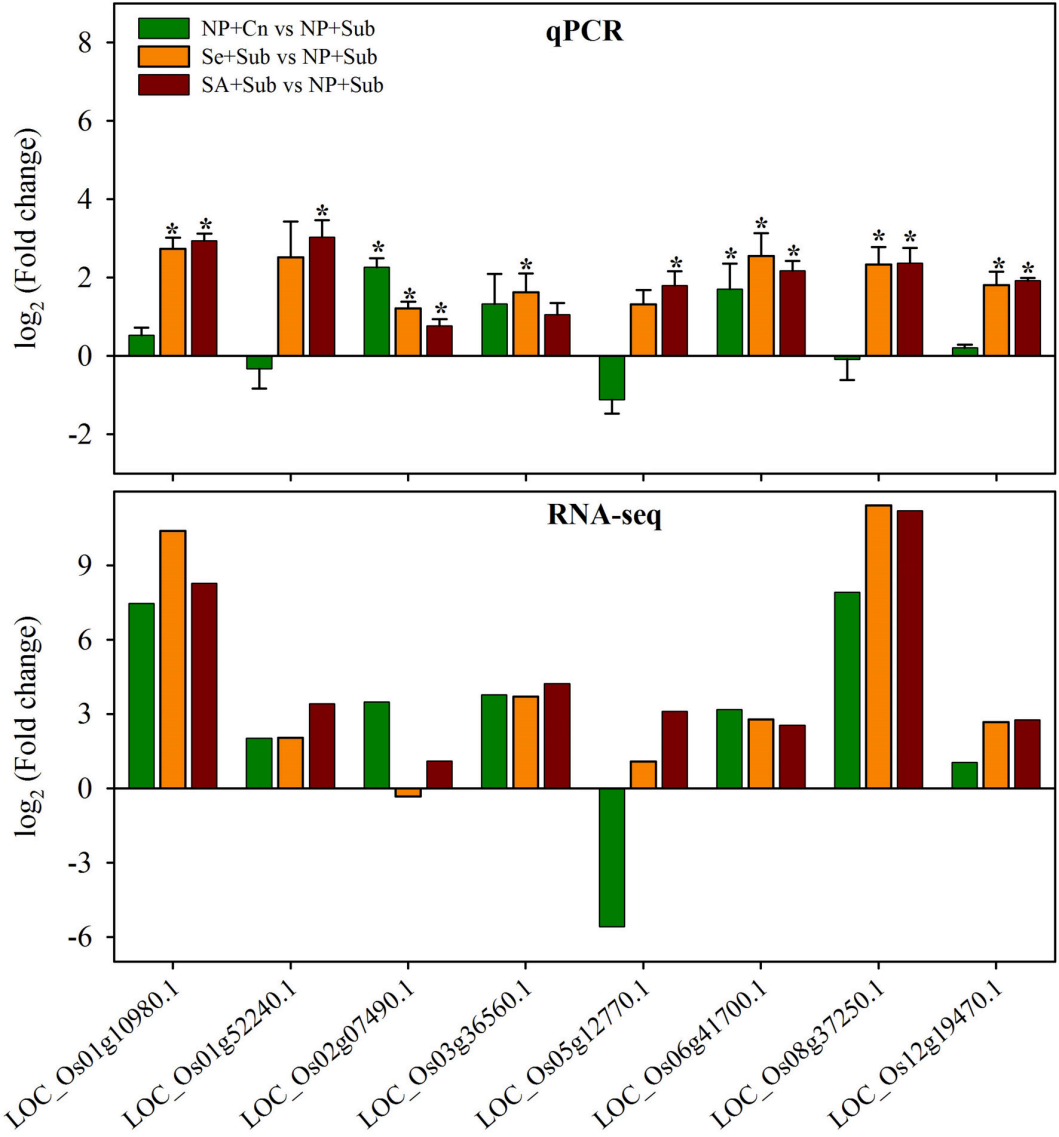
**Quantitative real-time PCR analysis of eight genes that were differentially expressed in NP+Cn, Se+Sub, and SA+Sub compared with NP+Sub**. Data represent mean ± *SE* from four independent biological replicates, and an asterisk (^*^) indicates a significant difference compared with NP+Sub (*P* < 0.05) according to Student's *t*-test. The genes comprised of LOC_Os01g10980.1 (development unspecified), LOC_Os01g52240.1 (chlorophyll a/b-binding protein), LOC_Os02g07490.1 (GAPDH: glyceraldehyde-3-phosphate dehydrogenase), LOC_Os03g36560.1 (POD: peroxidase), LOC_Os05g12770.1 (putative NBS-LRR-like protein), LOC_Os06g41700.1 (EXPA16: α-expansins), LOC_Os08g37250.1 (PLP2: patatin-like protein 2), and LOC_Os12g19470.1 (RBCS4: ribulose bisphosphate carboxylase small chain). The LOC_Os08g03290.1 (GAPDH) was kept as house-keeping gene.

To confirm the reliability of the RNA-Seq data, the expression of eight genes that were differentially expressed in NP+Cn, Se+Sub, and SA+Sub compared with NP+Sub, was assessed via qRT-PCR. The selected genes comprised of LOC_Os08g37250.1 (PLP2: patatin-like protein 2), LOC_Os01g10980.1 (development unspecified), LOC_Os05g12770.1 (putative NBS-LRR-like protein), LOC_Os02g07490.1 (GAPDH: glyceraldehyde-3-phosphate dehydrogenase), LOC_Os03g36560.1 (POD: peroxidase), LOC_Os01g52240.1 (chlorophyll a/b-binding protein), LOC_Os12g19470.1 (RBCS4: ribulose bisphosphate carboxylase small chain), and LOC_Os06g41700.1 (EXPA16: α-expansins). The LOC_Os08g03290.1 (GAPDH) was kept as house-keeping gene. Consistently, the results of the qRT-PCR assay exhibited the almost same trend to RNA-Seq data (Figure 6), confirming the reproducibility of RNA-Seq results. Results revealed (Figure 6) that all the genes were significantly (*P* ≤ 0.05) induced under of influence of both seed priming treatments except LOC_Os03g36560.1 (Se+Sub only), LOC_Os01g52240.1 (SA+Sub only), and LOC_Os05g12770.1 (SA+Sub only).

## Discussion

Due to several ecological and economic challenges in traditional transplanted rice systems, rice growers in Asian countries are shifting toward direct-seeding of rice. However, erratic germination and poor stand establishment of direct-seeded rice, particularly under unfavorable environmental conditions such as submergence stress, are major constraints for achieving optimal crop growth, and improved productivity. Seed priming has emerged as a tool for increasing the seed vigor, synchronization of germination, and seedling growth of rice under normal as well as stress conditions (Khaliq et al., 2015; Hussain et al., 2016a,b; Zheng et al., 2016).

The present study demonstrated that submergence stress severely hampered the germination and seedling growth of rice. However, both the seed-priming treatments were effective in alleviating the adverse effects of submergence stress (Figure 1). Under submergence stress, primed seeds exhibited higher germination, longer root and shoot, greater biomass accumulation, and seedling vigor index compared with non-primed seeds (Figure 1). Moreover, effects of seed priming were also apparent after desubmergence. Compared with non-primed seedlings, Se- and SA-primed rice seedlings exhibited better growth status and faster recovery (Figure S1). Previous studies conducted under aerated conditions have reported a positive role of seed priming under normal as well as under unfavorable environmental conditions including salinity, drought, chilling, and heavy metals (Bradford, 1986; Farooq et al., 2009; Khaliq et al., 2015; Zheng et al., 2016). In a study of the effect of seed priming on the flooding tolerance of rice, Ella et al. (2011) determined that seed treatments could substantially enhance crop establishment in flooded soils by enhancing antioxidant activity and carbohydrate metabolism. The early processes during germination (particularly cell division) are more sensitive to low oxygen stress, nevertheless, primed or pre-germinated seeds have the advantage of large carbohydrate storage reserves, which are helpful for their tolerance to low oxygen under flooded conditions (Ella et al., 2011). In the present study, higher germination and seedling growth of primed rice seeds were strongly associated with starch metabolism for both rice cultivars. Submergence stress severely reduced α-amylase activity and total soluble sugars in both rice cultivars (Figures 1G,H) by limiting starch degradation; therefore, seed reserves were not metabolized under submerged conditions. The ability of plants to degrade starch into soluble sugars plays a key role in their ability to survive and grow faster under a wide range of environments. In rice, amylase activity is highly induced during germination (Hussain et al., 2015, 2016a). In primed rice seeds, high α-amylase activity was reflected by higher soluble sugar concentrations (Figure 1) and a faster rate of starch breakdown in germinating primed seeds to provide the substrates necessary to generate the energy required for growth and maintenance processes. The degradation and conversion of seed reserves during germination may be related to the increase in soluble sugar contents in primed seedlings (Hussain et al., 2015). It is tempting to propose that priming does not simply accelerate germination-related processes but is involved in other specific mechanisms that improve germination and allow seeds to cope with environmental stresses during seedling establishment. Therefore, to further elucidate the mechanisms underlying priming-induced improvement of seed germination and growth under submergence stress, transcriptome analysis was performed using 4-days old seedlings. To control the variability in results due to individual differences, samples were pooled from at least 40 seedlings from each replicate. The results were further confirmed by qRT-PCR experiments for the eight selected genes with four biological replications of the RNA, including the same samples of RNA used in the transcriptome analysis. Comparative transcriptomic analysis of primed and non-primed rice seedlings under submergence stress identified 1811 and 1531 up-regulated, while 560 and 874 down-regulated transcripts as a consequence of Se+Sub and SA+Sub treatments, respectively (Figure 2). The total number of DETs in Se+Sub and SA+Sub was almost equal (Figure 2), which was consistent with the similar phenotypic performance of rice in these two treatments (Figure 1).

Pathway enrichment analysis indicated that the three pathways in Se+Sub (secondary metabolism, development, and cell) and four pathways in SA+Sub (development, transport, protein, and metal handling) were over-represented among DETs between primed and non-primed rice seedlings under submergence stress (Table 1). The enrichment of development-related genes due to seed priming treatments was consistent with the better germination and vigorous seedling growth of primed rice seedlings (Figure 1). Seed priming is well-known to trigger the pre-germinative metabolism; however, the effects may vary with species, priming reagents, priming duration, and environmental conditions (Farooq et al., 2009; Hussain et al., 2015). At the physiological level, early seed imbibition and the seed repair response (activation of DNA repair pathways, and antioxidant mechanisms) occur, which are essential to preserve genome integrity, ensuring proper germination, and seedling development (Chen and Arora, 2013; Paparella et al., 2015). In the past, the involvement of some processes during seed priming, such as cell cycle-related events (De Castro et al., 2000), endosperm weakening by hydrolase activities (Groot et al., 1988; Bradford et al., 2000), and mobilization of storage proteins (Job et al., 1997; Gallardo et al., 2001) had been described.

The germination process of any crop relies on the kinetics of imbibition, reserve mobilization, cell elongation and division, and management of secondary stresses such as oxygen deprivation (Magneschi and Perata, 2009; Miro and Ismail, 2013). Under submergence stress, various specific expansins play important roles in coleoptile elongation because of their action in cell wall loosening (Huang et al., 2000; Choi et al., 2003; Magneschi and Perata, 2009). Several expansins, including *EXPA2* (Huang et al., 2000), *EXPA4* (Huang et al., 2000; Choi et al., 2003), *EXPA7, EXPB12* (Lasanthi-Kudahettige et al., 2007), *EXPA1, EXPB11*, and *EXPB17* (Takahashi et al., 2011), have been reported to be linked with coleoptile elongation under submergence stress. Consistently, in the present study, enhanced expression of expansins genes (e.g., *EXPA7* and *EXPA16*) genes was observed with both seed priming treatment compared with NP+Sub. The cell function overview pathway revealed that most up-DETs between seed priming treatments (particularly Se+Sub) and NP+Sub were involved in cell division and cell cycle (Figure 3). Germination process is based on coordinated cell elongation and division; therefore, it is not surprising that α-expansin proteins, which are associated with cell division, were strongly up-regulated due to seed priming (Lee et al., 2001; Lee and Kende, 2002).

Cell pathway and metal handling pathway showed the enrichments in Se and SA priming treatments, respectively (Table 1), suggesting their specific role in these pathways. Whiling reviewing a number of studies, Feng et al. (2013) concluded that Se maintains cell structure and strengthen the cell integrity under various abiotic stresses, including salinity, water, chilling, and heavy metals. Moreover, optimal supplementation of Se decreases the ROS generation and diminishes the damages to the lipids of the plant cell membranes. Likewise, protective role of SA particularly when applied exogenously against heavy metals is summarized by Hayat et al. (2010).

The GO enrichment analysis also depicted that transcripts that function in regulation of energy metabolism, photosynthesis, defense response, protein modification process, response to chemical stimulus, regulation of transcription, and metabolic processes were up-regulated by seed priming treatments (Figure 4; Table S6). However, some genes related to protein ubiquitination, regulation of transcription, regulation of primary metabolism, signal transmission, and response to stress were down-regulated compared with NP+Sub (Figure 5). Those genes modulated by seed priming treatments might also have contributed to priming-induced submergence tolerance in rice. Several researchers in the past have documented that seed priming regulates many germination-related events such as respiration and energy metabolism, gene transcription and translation, and early reserve mobilization (Varierf et al., 2010; Chen and Arora, 2013; Paparella et al., 2015). Limitations of energy supply under submergence-induced anaerobic conditions are a major bottleneck for better seed germination and seedling growth (Miro and Ismail, 2013). However, early imbibition process due to priming promotes the efficient mitochondrial development by augmenting energy metabolism, and therefore, the primed seeds with a greater ATP pool and a potentially more efficient ATP-producing system are better equipped for post-priming germination (Chen and Arora, 2013). Moreover after rehydration of primed seeds, main cellular processes such as the *de-novo* synthesis of nucleic acids and proteins, ATP production, accumulation of sterols and phospholipids, activation of DNA repair, and antioxidant mechanisms are triggered leading to higher stress tolerance ability (Varierf et al., 2010; Chen and Arora, 2013).

Submergence tolerance of rice seedling is related to the maintenance of energy supply, which requires high levels of carbohydrates (Miro and Ismail, 2013). Our study revealed that the many genes involved cellular and metabolic processes such as carbohydrate metabolism, nitrogen compound metabolic process, cellular and metabolic biosynthesis, transcription, and response to oxidative stress were highly up-regulated and overlapped in seed priming treatments compared with NP+Sub (Tables S3, S6). The enrichment of various pathways related to carbohydrate metabolism (e.g., starch degradation, trehalose biosynthesis, anaerobic respiration) due to seed priming was consistent with higher concentrations of soluble sugars in these treatments (Figure 1H). The sugars, sucrose, and glucose either act as substrates for cellular respiration or act as osmolytes to maintain cell homeostasis. In submerged conditions, glycolysis and alcoholic fermentation are important for the energy production of plants. In present study, the expressions of the genes involved in the energy production (e.g., GAPDH, NADH-GOGAT) were highly induced under the influence of seed priming treatments (Figure 6; Tables S2, S3). Previously, it has been reported that the induction of these genes was correlated with anaerobic tolerance (Umeda and Uchimiya, 1994; Sachs et al., 1996). Tamura et al. (2011) also stated that the induced expression of NADH-GOGAT genes was concomitant with enhanced biomass accumulation in rice.

Submergence stress is known to enhance the accumulation of ROS, which can disrupt the normal functioning of the plants and cause oxidative stress (Blokhina and Fagerstedt, 2010; Gill and Tuteja, 2010). The ability of plant to manage ROS detoxification can influence its survival in submerged conditions. In present study, many genes involved in antioxidative system of the plants (e.g., PEROXIDASE) were regulated by seed priming (Figure 6; Table S2), which are known to negate the ROS-induced effects and to overcome the oxidative stress (Gill and Tuteja, 2010). Previously, many studies have reported the enhanced antioxidative defense system of primed seedlings under stressful conditions (Jisha et al., 2013; Zheng et al., 2016). In an integrated transcriptomic and proteomic study, Kubala et al. (2015) demonstrated that the osmopriming positively regulated the proteins involved in the management of oxidative stress (e.g., CAT, POX) during germination. Moreover, Bailly et al. (2000) also reported that the activities of detoxifying enzymes such as SOD, POD, and CAT, were increased in response to seed priming.

It was also found that various transcripts related to proteins were altered under the influence of Se- and SA-priming. Regulation of storage proteins (e.g., *PLP2*) due to seed priming treatments (Figure 6; Tables S2, S3, S5) is in accordance with Job et al. (1997). Similar behavior of storage proteins during seed priming of *Arabidopsis* (Gallardo et al., 2001) and sugarbeet (Catusse et al., 2011) was observed in proteome studies, which suggest the similarity of different plant species concerning mobilization of storage proteins during priming. Gallardo et al. (2001) and Catusse et al. (2011) also reported the seed priming-induced alterations in several other proteins involved in different biological processes including cell cycle components, enzymes of glyoxylate cycle, methionine biosynthesis, translation initiation factors, and ABA signaling elements. A transcriptome study further indicated the gene expression associated with translation and protein functioning, such as protein synthesis, cell cycle, and carbon metabolisms (Soeda et al., 2005).

Rubisco protein plays an important role in carbon dioxide (CO_2_) fixation and oxidative fragmentation of the pentose substrate. Mommer et al. (2005) reported a key role of Rubisco under submergence stress, in which the carboxylation capacity was decreased. In the present study, rice seedlings also exhibited low levels of Rubisco protein (*RBCS4*) in NP+Sub (Figure 6; Tables S2, S3), which could lead to lower levels of carboxylation capacity in a manner similar to that proposed by Mommer et al. (2005). Both seed-priming treatments effectively enhanced the expression of Rubisco protein, suggesting that seed priming may trigger the CO_2_ fixation and oxidative fragmentation of the pentose substrate.

*Chl a/b*-binding protein is the major protein component of chloroplast thylakoid membranes and harvests light energy for photosynthesis (Figure 6; Tables S2, S3). Submergence stress severely inhibited the expression of *Chl a/b*; therefore, the seedlings in NP+Sub did not attain a dark green color (Figure 1). This response was similar to that observed in geminivirus infection, in which light-harvesting complexes were down-regulated at the mRNA level in *Arabidopsis* (Ascencio-Ibanez et al., 2008). However, seed priming with Se and SA effectively enhanced the expression of *Chl a/b*, consistent with the dark green color of the seedlings in Se+Sub and SA+Sub (Figure 1). This highlights that the *Chl a/b* could be a key factor for efficient photosynthesis to maintain energy metabolism for plant growth under submergence stress.

In rice, cell death and subsequent lysis triggers the formation of aerenchyma, which may play a key role in submergence tolerance by facilitating internal aeration (Jackson, 1985; Nishiuchi et al., 2012). Aerenchyma and leaf gas films in submerged leaves enhance the exchange of O_2_ and CO_2_ between leaves and the surrounding water, and thus promote underwater net photosynthesis by supplying CO_2_ during the day time and increase O_2_ uptake for respiration at night (Colmer and Voesenek, 2009; Pedersen et al., 2009). Generally, aerenchyma formation occurs after 24–72 h of anaerobic treatment (Haque et al., 2010; Rajhi et al., 2011; Nishiuchi et al., 2012) and is enhanced with seedling elongation and ethylene (Steffens et al., 2011). Interestingly, our results depicted that genes involved in cell death (e.g., putative NBS-LRR-like protein) and photosynthesis were up-regulated in SA+Sub (Figure 4; Tables S2, S3), suggesting that SA might have a possible role in aerenchyma formation under submerged conditions. However, further investigations are required to find the exact role of SA and the genetic regulation of lysigenous aerenchyma formation.

Submergence-induced ERFs play a dynamic role in the stress tolerance of plants. Ethylene triggers the expression of the *Sub1A* gene; however, this mechanism is not usually active for the germination of rice under submergence stress. Xu et al. (2006) demonstrated that rice cultivars lacking the *Sub1A* exhibited higher germination under anoxia. This finding does not rule out the possibility that other *ERFs* may play a role in submergence tolerance at the germination stage. In the present study, seed priming treatments were found to modulate several *ERFs* such as *ERF47, ERF108, ERF35*, and *ERF20, ERF79* (Table S2). These *ERFs* belong to the same clade as *Sub1A* or Arabidopsis group *VII ERF* (*HRE1, HRE2*), which are up-regulated under hypoxic conditions and protect *Arabidopsis* plants from anoxic stress (Licausi et al., 2010). Regulation of these *ERFs* due to seed priming (Table S2) might contribute to the enhanced submergence tolerance of primed-seedlings. In crux, seed priming triggered extensive transcriptional reprogramming and pre-conditioned resistance to submergence stress. Further investigation of the *in vivo* roles of these genes will shed additional light on seed priming-induced submergence tolerance in rice.

The results provided in this study offer an initial step toward the identification of genes and expand our understanding of the complex mechanisms involved in seed priming-induced submergence tolerance in rice. Functional analyses revealed that the genes involved in regulation of secondary metabolism, development, cell, transport, protein, and metal handling were over-represented after Se- or SA priming. These coordinated factors enhanced the tolerance of rice to submergence stress and maintained the germination and seedling growth of rice. A number of identified transcripts in present study had previously been shown to play a role in submergence tolerance of several plant species, a finding that underlines the usefulness of seed priming techniques for rice plantation in in submergence-prone areas. Moreover, the present study guided toward some new pathways/transcripts associated with seed priming, the role of identified genes in specific pathways should be validated in further studies.

## Author contributions

SH, SP, and LN: initiated and designed the research; SH, HY, and FK: performed the experiments; SH, HY, FK, and MS: analyzed the data; HH and FK: annotated the RNA-Seq data; SH, HY, and LN: wrote the manuscript; JH and KC: assisted with manuscript preparation. All of the authors discussed the results and commented on the manuscript.

## Funding

This work is supported by the National Natural Science Foundation of China (Project No. 31371571), and the Fundamental Research Funds for the Central Universities (Project No. 2013PY109).

### Conflict of interest statement

The authors declare that the research was conducted in the absence of any commercial or financial relationships that could be construed as a potential conflict of interest.

## Supplementary material

Figure S1 **Pictorial view of the primed and non-primed rice seedlings after 4 days of desubmergence**. Desubmergence was done at 8 DAS. NP, non-primed; Se, 60 μM selenium priming; SA, 100 mg L^−1^ salicylic acid priming; Sub, submergence. Submergence stress (Sub) was imposed by adding 13 cm of distilled water in each plastic cups, while wet filter paper was used for the NP+Cn treatment. After desubmergence (8 DAS), a 20 ml of nutrient solution containing 1 mM (NH_4_)_2_SO_4_, 1 mM NaH_2_PO_4_, 1 mM KCl, 1 mM Ca(NO_3_)_2_, 2 mM Na_2_SiO_3_.9H_2_O, 1 mM MgSO_4_.7H_2_O, 10 μM H_3_BO_3_, 1 μM ZnSO_4_.7H_2_O, 0.5 μM MnSO_4_.H_2_O, 0.1 μM CuSO_4_.5H_2_O, 0.05 μM (NH_4_)6Mo_7_O_24_.4H_2_O, and 20 μM FeNa-EDTA, was added to each plastic cup. Both the seed priming treatments showed faster recovery and better growth compared with NP+Sub.

Table S1 **List of specific primers used for qRT-PCR analysis**. F and R denote the forward and reserve primers, respectively.

Table S2 **List of DETs in NP+Cn (A), Se+Sub (B), and SA+Sub (C) treatments compared with NP+Sub**. Genes with significantly (Fold change > |2|, *FDR* < 0.05) increased and decreased expression are presented.

Table S3 **A list of 1628 DETs overlapped in seed priming treatments (Se+Sub and SA+Sub) compared with NP+Sub**.

Table S4 **Top 10 enriched common regulatory elements from 1628 DETs promoters (fold change > |2|, *FDR* < 0.05) due to seed priming treatments (Se+Sub and SA+Sub)**.

Table S5 **List of DETs associated with development pathway in NP+Cn (A), Se+Sub (B), and SA+Sub (C) treatments**. Based on pathway enrichment analysis by Mapman, a total of 131, 93, and 95 development-related genes were overrepresented in NP+Cn, Se+Sub, and SA+Sub treatments respectively, compared with NP+Sub.

Table S6 **Overlapped GO enrichments in seed priming treatments (Se+Sub and SA+Sub) compared with NP+Sub**.
