# Supplementary figures and images for: Comparative Transcriptional Profiling of Primed and Non-primed Rice Seedlings under Submergence Stress
Source: Front Plant Sci. 2016 Jul 28;7:1125. doi: 10.3389/fpls.2016.01125 (PMC4964843; doi:10.3389/fpls.2016.01125)

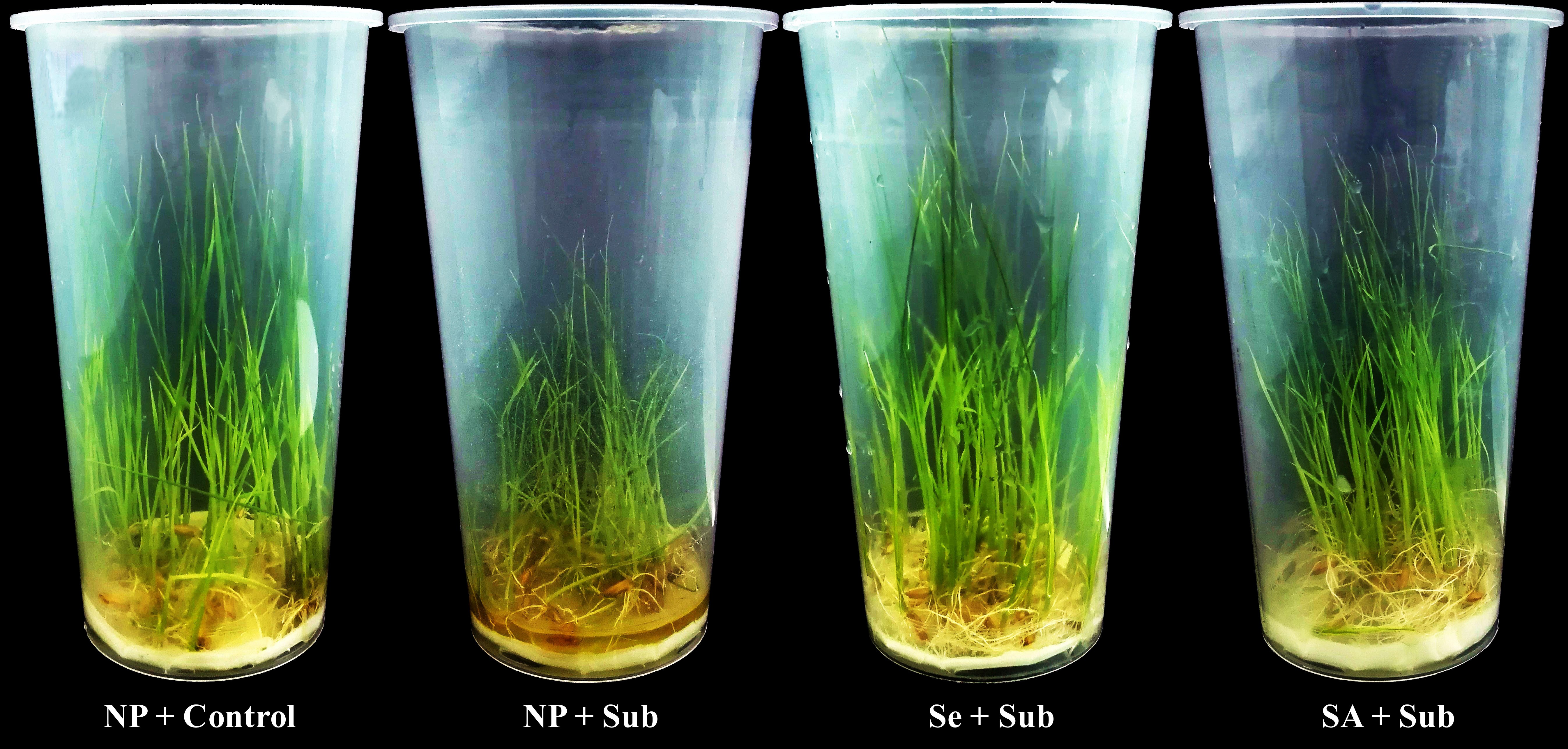

Supplement: Figure S1 — Pictorial view of the primed and non-primed rice seedlings after 4 days of desubmergence. Desubmergence was done at 8 DAS. NP, non-primed; Se, 60 μM selenium priming; SA, 100 mg L−1 salicylic acid priming; Sub, submergence. Submergence stress (Sub) was imposed by adding 13 cm of distilled water in each plastic cups, while wet filter paper was used for the NP+Cn treatment. After desubmergence (8 DAS), a 20 ml of nutrient solution containing 1 mM (NH4)2SO4, 1 mM NaH2PO4, 1 mM KCl, 1 mM Ca(NO3)2, 2 mM Na2SiO3.9H2O, 1 mM MgSO4.7H2O, 10 μM H3BO3, 1 μM ZnSO4.7H2O, 0.5 μM MnSO4.H2O, 0.1 μM CuSO4.5H2O, 0.05 μM (NH4)6Mo7O24.4H2O, and 20 μM FeNa-EDTA, was added to each plastic cup. Both the seed priming treatments showed faster recovery and better growth compared with NP+Sub. [file Image1.JPEG]
