# Supplementary material for: Comparative Transcriptional Profiling of Primed and Non-primed Rice Seedlings under Submergence Stress
Source: Front Plant Sci. 2016 Jul 28;7:1125. doi: 10.3389/fpls.2016.01125 (PMC4964843; doi:10.3389/fpls.2016.01125)
Supplement: Table S4 — Top 10 enriched common regulatory elements from 1628 DETs promoters (fold change > |2|, FDR < 0.05) due to seed priming treatments (Se+Sub and SA+Sub). [file DataSheet4.DOC]

**Table S4: Top 10 enriched common regulatory elements from 1628 DETs promoters (Fold change > |2|, FDR<0.05) due to seed priming treatments.**

| **Common Motif** | **Gene Num*** | **% in genes** | **Motif logo** | **Regulatory elements** |
| --- | --- | --- | --- | --- |
| **TATANBDAC** | 1477  (1163/314) | 90.7 | 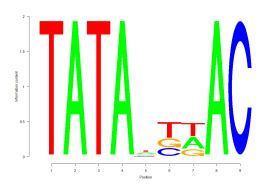 | TATA box /  MADS box factors |
| **GCGAST** | 1219  (953/266) | 74.9 | 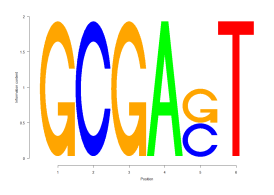 | Basic helix-loop-helix factors (bHLH) |
| **TNNCCAVCG** | 818  (632/186) | 50.2 | 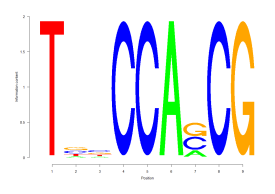 | Basic helix-loop-helix factors (bHLH) |
| **GCACANAT** | 649  (490/159) | 39.9 | 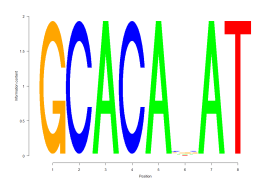 | CAAT box /  AP2/ERF domain |
| **CGCGGA** | 636  (499/137) | 39.1 | 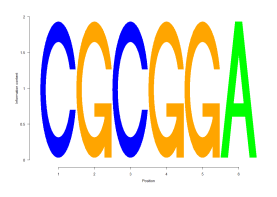 | CG-1 domain |
| **GDGCGGHGH** | 610  (451/159) | 37.5 | 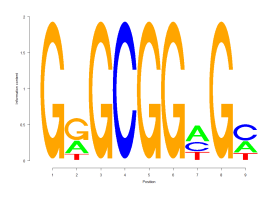 | GC box /  AP2/ERF domain |
| **AHATAATAA** | 558  (445/113) | 34.3 | 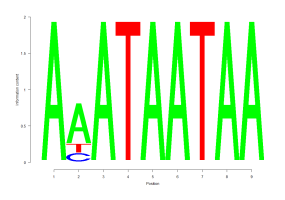 | MADS box factors |
| **GCGGNGCG** | 507  (402/105) | 31.1 | 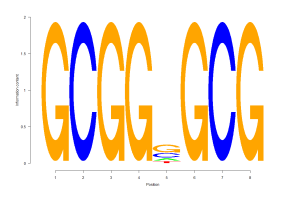 | GC box /  AP2/ERF domain |
| **TNCGCGNDG** | 481  (363/118) | 29.5 | 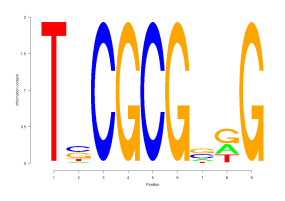 | Tryptophan cluster factors |
| **GNNDCTCGCS** | 381  (299/82) | 23.4 | 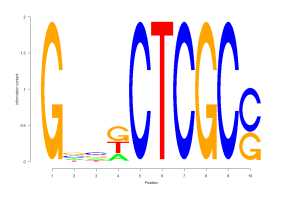 | Basic leucine zipper factors (bZIP) |

- In brackets, nominator and denominator represent up-regulated and down-regulated gene number, respectively.
